# Comparison of early warning scores for predicting clinical deterioration and infection in obstetric patients

**DOI:** 10.1186/s12884-022-04631-0

**Published:** 2022-04-06

**Authors:** David E. Arnolds, Kyle A. Carey, Lena Braginsky, Roxane Holt, Dana P. Edelson, Barbara M. Scavone, Matthew Churpek

**Affiliations:** 1 University of Chicago, Chicago, IL USA; 2 University of Wisconsin-Madison, Madison, WI USA

**Keywords:** Early warning systems, Maternal morbidity, Machine learning

## Abstract

**Background:**

Early warning scores are designed to identify hospitalized patients who are at high risk of clinical deterioration. Although many general scores have been developed for the medical-surgical wards, specific scores have also been developed for obstetric patients due to differences in normal vital sign ranges and potential complications in this unique population. The comparative performance of general and obstetric early warning scores for predicting deterioration and infection on the maternal wards is not known.

**Methods:**

This was an observational cohort study at the University of Chicago that included patients hospitalized on obstetric wards from November 2008 to December 2018. Obstetric scores (modified early obstetric warning system (MEOWS), maternal early warning criteria (MEWC), and maternal early warning trigger (MEWT)), paper-based general scores (Modified Early Warning Score (MEWS) and National Early Warning Score (NEWS), and a general score developed using machine learning (electronic Cardiac Arrest Risk Triage (eCART) score) were compared using the area under the receiver operating characteristic score (AUC) for predicting ward to intensive care unit (ICU) transfer and/or death and new infection.

**Results:**

A total of 19,611 patients were included, with 43 (0.2%) experiencing deterioration (ICU transfer and/or death) and 88 (0.4%) experiencing an infection. eCART had the highest discrimination for deterioration (*p* < 0.05 for all comparisons), with an AUC of 0.86, followed by MEOWS (0.74), NEWS (0.72), MEWC (0.71), MEWS (0.70), and MEWT (0.65). MEWC, MEWT, and MEOWS had higher accuracy than MEWS and NEWS but lower accuracy than eCART at specific cut-off thresholds. For predicting infection, eCART (AUC 0.77) had the highest discrimination.

**Conclusions:**

Within the limitations of our retrospective study, eCART had the highest accuracy for predicting deterioration and infection in our ante- and postpartum patient population. Maternal early warning scores were more accurate than MEWS and NEWS. While institutional choice of an early warning system is complex, our results have important implications for the risk stratification of maternal ward patients, especially since the low prevalence of events means that small improvements in accuracy can lead to large decreases in false alarms.

**Supplementary Information:**

The online version contains supplementary material available at 10.1186/s12884-022-04631-0.

## Background

Maternal morbidity and mortality are increasing in the United States [1–3]. In 1987, there were 7.2 pregnancy-related deaths per 100,000 live births, which increased to 16.9 by 2016 [1]. Severe maternal morbidity has also increased, which includes a rise in pregnancy-related hospitalizations [2, 3]. Studies of severe maternal morbidity and mortality suggest that many cases of maternal morbidity and mortality are preventable, with errors and delays in diagnosis and treatment contributing to preventable events [4, 5]. Recognition of this has resulted in efforts to formalize criteria to identify pregnant or postpartum women who may be at risk for adverse outcomes, and the Council on Patient Safety in Women’s Healthcare recommends widespread adoption of such practices [6–8].

A number of early warning systems have been proposed to identify hospitalized patients at risk for clinical deterioration [6–13]. These systems vary in the parameters examined, cutoffs considered to be abnormal, and complexity in scoring. Scoring systems that have been developed for use in the general medical and surgical population, such as the Modified Early Warning Score (MEWS) and national early warning score (NEWS) [10, 11], have been applied to pregnant and post-partum patients, although recognition of the wide range of vitals that occur in normal pregnancy has also led to pregnancy-specific scoring systems, such as the modified early obstetric warning system (MEOWS), maternal early warning criteria (MEWC), and maternal early warning trigger (MEWT) [6, 9, 14, 15]. Attempts to validate some of these pregnancy-specific systems have yielded mixed results depending on the setting, definition of morbidity used, and accuracy of metrics studied [7, 8, 15–18]. A more recent development is the use of statistical modeling that continuously calculates a risk score based on data present in the electronic health record; this is the basis for the electronic Cardiac Arrest Risk Triage (eCART) score, which has been validated in the general medical and surgical populations [13, 19, 20]. However, the eCART score has not previously been evaluated on antepartum or postpartum wards.

Therefore, we aimed to evaluate the performance of MEWS and NEWS, which are commonly used scoring systems developed originally for the general medical-surgical population [10, 11], the maternity-specific MEOWS, MEWC, and MEWT scores, and eCART on the antepartum and postpartum floors [6, 9, 13, 15, 20]. Our primary outcome was a composite of death or intensive care unit (ICU) admission. We also evaluated the performance of the algorithms in detecting infection as a marker for clinically significant deterioration because death or ICU admission are both rare in the obstetric population.

## Methods

### Study population and data collection

We conducted a retrospective cohort study of all adult (age ≥ 18 years) patients admitted to a hospital ward following transfer from labor and delivery at the University of Chicago Medicine from November 2008 to December 2018. The cohort includes both postpartum patients as well as patients who were initially admitted to labor and delivery prior to transfer to the antepartum ward. Patient demographic information as well as time- and location-stamped vital sign and laboratory results were obtained from electronic health record data (Epic; Verona, WI).

### Outcomes

The primary study outcome was death or transfer to the ICU. ICU transfer was defined as going directly from the antepartum or postpartum ward to the ICU or going from the ward to labor and delivery and then directly to an ICU within 24 h. During the study time frame, all patients requiring invasive ventilatory support or vasoactive infusions were cared for in the ICU. In addition, patients thought to be at risk for hemodynamic collapse or respiratory failure could be transferred to the ICU for more intensive monitoring and therapy at the discretion of the attending physician. We did not otherwise include direct transfers from labor and delivery to the ICU, as our study was focused on the evaluation of early warning systems in the ward setting. The secondary outcome was the development of a new infection, which was defined by the administration of intravenous (IV) antibiotics within 2 days before or after a blood culture order followed by four consecutive days of IV and/or oral antibiotics or up to the day before discharge, as previously published by Rhee et al [21] and which we found in our prior work to be the most specific health record criteria for identifying infections [22]. While antibiotic administration during the study timeframe was ultimately at the discretion of the treating physician, institutional guidelines typically recommended at least 7 days of appropriate antibiotics following a positive blood culture.

### Early warning scores

We evaluated the performance of early warning scores developed for general medical-surgical patients (MEWS, NEWS, and eCART) and specifically for pregnant and post-partum patients (MEOWS, MEWC, MEWT). These tools have been previously described and are summarized in Additional File 1. MEWS and NEWS are commonly used general aggregate weighted scores where increasing scores denote a higher risk of deterioration [10, 11]. A random forest version of eCART was used in this study, which is a previously derived model that combines thousands of individual decision trees into a model that outputs the probability of clinical deterioration in the following eight hours (ICU transfer, cardiac arrest, or death) in a cohort of general medical-surgical ward patients [20]. Notably, this version of eCART was directly applied to the current study without alteration in order to test the ability of this general score to identify deterioration in the obstetric population. MEOWS thresholds were the same as used by Singh et al., with a trigger defined as a single markedly abnormal observation (red trigger) or two simultaneous mildly abnormal observations (two yellow triggers) [15] MEWT was calculated based on the work by Shields et al., which similarly requires either two less severe triggers or one severe trigger [9]. MEWC is a single parameter score whereby any abnormal value beyond the variable thresholds results in a trigger [6]. All of these tools incorporate vital signs with varying thresholds denoting abnormality, and eCART additionally includes laboratory values, age, and prior ICU stay. Given the nature of our study we were unable to include subjective parameters (ie nursing discomfort with status, or headache in a patient with pre-eclampsia) that are included in the MEOWS, MEWT, and MEWC.

### Statistical analysis

Patient characteristics between those who experienced and did not experience the primary outcome were compared using t-tests, Wilcoxon rank-sum tests, and chi-squared tests, as appropriate. Model discrimination was calculated using the area under the receiver operating characteristic curve (AUC) by calculating the score at each observation time and looking forward to see if the outcome occurred within 24 h of each observation time. AUCs were compared using the method Delong [23]. All analyses were performed using Stata version 15.1 with a two-sided *p* < 0.05 denoting statistical significance.

## Results

A total of 19,611 patients were admitted to labor and delivery and subsequently transferred to our antepartum or postpartum ward and are included in the analysis. A study flowchart describing the identification of patients included in the analysis is provided in Additional File 2. Forty-three women died or were admitted to the ICU within 24 h of a ward observation (0.2%), which included three deaths. Two additional deaths occurred more than 24 h after any ward observations. Eighty-eight women (0.4%) met criteria for infection within 24 h of a ward observation. Patient characteristics are described in Table 1, with comparisons between patients who did and did not experience the primary outcome (ward to ICU transfer and/or death). No differences in age, ethnicity, or body mass index were identified in women who died or were transferred to the ICU compared with those who did not. Women experiencing the primary outcome were more likely than those who did not to have a hypertensive disorder (27.9% vs. 5.6%; *p* < 0.001) or diabetes mellitus (9.3% vs. 2.1%; *p* = 0.01). Women experiencing the primary outcome had a longer total length of stay (median 8, IQR 6–12 days) compared to women not experiencing the primary outcome (median 3, IQR 2–3 days; *p* < 0.01).

**Table 1:** Comparisons of patient characteristics between patients who did and did not experience an ICU transfer and/or death

	No ICU transfer or death (*n* = 19,568)	ICU transfer or death (*n* = 43)	*P value*
Age, years (median, IQR^a^)	27 (23, 32)	29 (24, 33)	0.11
Race/ethnicity (n, %)			0.85
Black	14,586 (74.5%)	32 (74.4%)	
White	2,266 (11.6%)	4 (9.3%)	
Hispanic	1,335 (6.8%)	4 (9.3%)	
Other/Unknown	1,381 (7.1%)	3 (7.0%)	
Body Mass Index (kg/m^2^) at admission (median, IQR)	31.3 (27.0, 37.0)	32.6 (26.2, 39.2)	0.59
Diabetes Mellitus (n, %)	412 (2.1%)	4 (9.3%)	0.01
Hypertensive disorders (n, %)	1,102 (5.6%)	12 (27.9%)	< 0.001
Total length of stay, days (median, IQR)	3 (2, 3)	8 (6, 12)	< 0.001
In-Hospital mortality (n, %)	2 (0.01%)	3 (7.0%)	< 0.001

^a^ Interquartile range

**Fig. 1:**
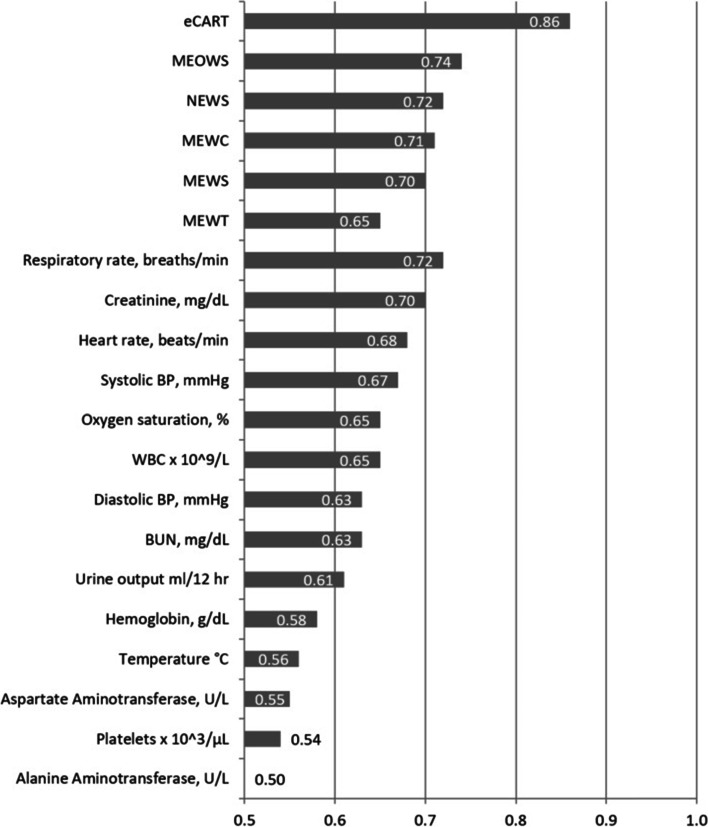
AUCs of early warning scores and individual variables for predicting ICU transfer and/or death

Distributions of the different scores and physiological data in the dataset are shown in Table 2, stratified by patients with and without the primary outcome. As shown, scoring system values were generally higher, with vital signs and laboratory values more abnormal for those patients who died or were transferred to the ICU, although average values were mostly in the normal range for both groups. The performance of each scoring system, as well as the component vital signs and laboratory values for the primary outcome of ICU admission or death is shown in Fig. 1.

**Table 2:** Early warning score and individual variable distributions in the cohort

	No ICU transfer or death (*n* = 19,568)	ICU transfer or death (*n* = 43)	*p*-value
MEWS^a^	1 (1, 2)	1 (1, 2)	< 0.001
NEWS^b^	1 (0, 2)	2 (1, 4)	< 0.001
eCART^c^	2 (2, 3)	4 (3, 7)	< 0.001
MEOWS^d^	0 (0, 0)	0 (0, 1)	< 0.001
MEWC^e^	0 (0, 0)	0 (0, 1)	< 0.001
MEWT^f^	0 (0, 0)	0 (0, 0)	< 0.001
Respiratory rate (breaths per minute)	18 (18, 20)	18 (18, 20)	< 0.001
Heart rate (beats per minute)	85 (75, 94)	92 (82, 105)	< 0.001
Temperature (°C)	36.6 (36.3, 36.8)	36.6 (36.3, 36.9)	< 0.001
WBC^g^ (× 10^3^/uL)	11.6 (9, 14.8)	13.1 (8.7, 16.6)	< 0.001
BUN^h^ (mg/dL)	8 (5, 11)	10 (8, 15)	< 0.001
Creatinine (mg/dL)	0.6 (0.5, 0.8)	0.7 (0.6, 1)	< 0.001
AST^i^ (U/L)	25 (17, 40)	29 (21, 48)	< 0.001
ALT^j^ (U/L)	20 (11, 48)	27 (14, 54)	< 0.001
Hemoglobin (g/dL)	9.8 (8.7, 10.8)	8.6 (7.6, 9.8)	< 0.001
Platelet count (× 10^3^/uL)	203 (154, 259)	209.5 (155, 347)	< 0.001
Systolic Blood Pressure (mm Hg)	117 (107, 129)	127 (112, 144)	< 0.001
Diastolic Blood Pressure (mm Hg)	67 (60, 76)	74 (63, 85)	< 0.001
Oxygen saturation (%)	98 (97, 99)	98 (96, 99)	< 0.001
Urine output (mL/12 h)^k^	0 (0, 855)	717.5 (2, 1300)	< 0.001

^a^ Modified early warning score

^b^ National early warning system

^c^ electronic cardiac arrest triage

^d^ Modified early obstetric warning system

^e^ Maternal early warning criteria

^f^ Maternal early warning trigger

^g^ White blood cell count

^h^ Blood urea nitrogen

^i^ Aspartate amniotransferase

^j^ Alanine transaminase

^k^ Unquantified urine was recorded as 0

**Fig. 2:**
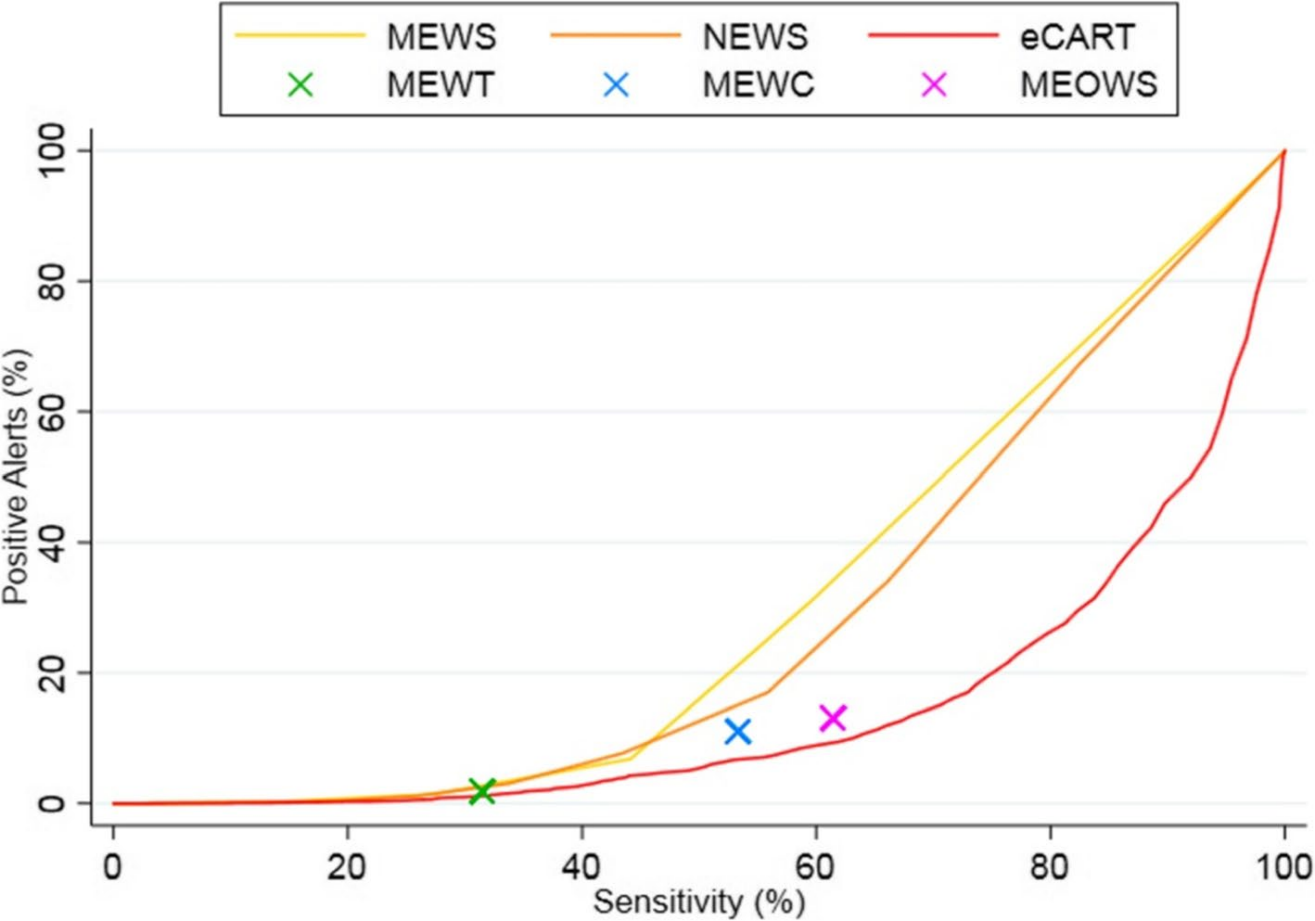
Early warning score efficiency curve illustrating sensitivity (x-axis) versus the percentage of observations meeting a given threshold (positive alerts; y-axis). As shown, eCART is the most efficient score (highest sensitivity for a given number of positive alerts), followed by the obstetric scores, and then the commonly used general scores

eCART had the highest discrimination for the primary outcome (*p* < 0.05 for all comparisons), with an AUC of 0.86 (95% CI 0.84–0.87), followed by MEOWS (0.74 (95% CI 0.72–0.76), NEWS (0.72 (95% CI 0.70–0.75), MEWC (0.71 (95% CI 0.69–0.73), MEWS (0.70 (95% CI 0.67–0.72), and MEWT (0.65 (95% CI 0.63–0.67). Respiratory rate had the highest AUC among the individual variables (AUC 0.72 (95% CI 0.70–0.74), followed by creatinine (0.70 (95% CI 0.68–0.73), heart rate (0.68 (95% CI 0.65–0.71), and systolic blood pressure (0.67 (95% CI 0.65–0.70). The sensitivity, specificity, positive, and negative predictive values for each scoring are shown in Additional File 3. As shown, eCART had higher accuracy compared to the general early warning scores across different thresholds. For example, an eCART score ≥ 0.006 had a sensitivity of 41% at a specificity of 97%, whereas NEWS ≥ 5 had a 34% sensitivity and MEWS ≥ 4 had a 28% sensitivity at a similar specificity. The maternal early warning scores also had higher accuracy at specific thresholds than MEWS or NEWS. For example, MEWC had a sensitivity of 53% with a specificity of 89% compared to NEWS ≥ 4 with a sensitivity of 43% and specificity of 92%. MEOWS had a sensitivity of 61% and a specificity of 87%, while MEWT was less sensitive (31%) but more specific (98%) than the other scores. These data are illustrated using early warning score efficiency curves (Fig. 2), which shows the percentage of observations that would trigger an alert at each threshold versus that threshold’s sensitivity. eCART was the most efficient score, followed by the obstetric scores, and then the commonly used general scores (MEWS and NEWS).

**Fig. 3:**
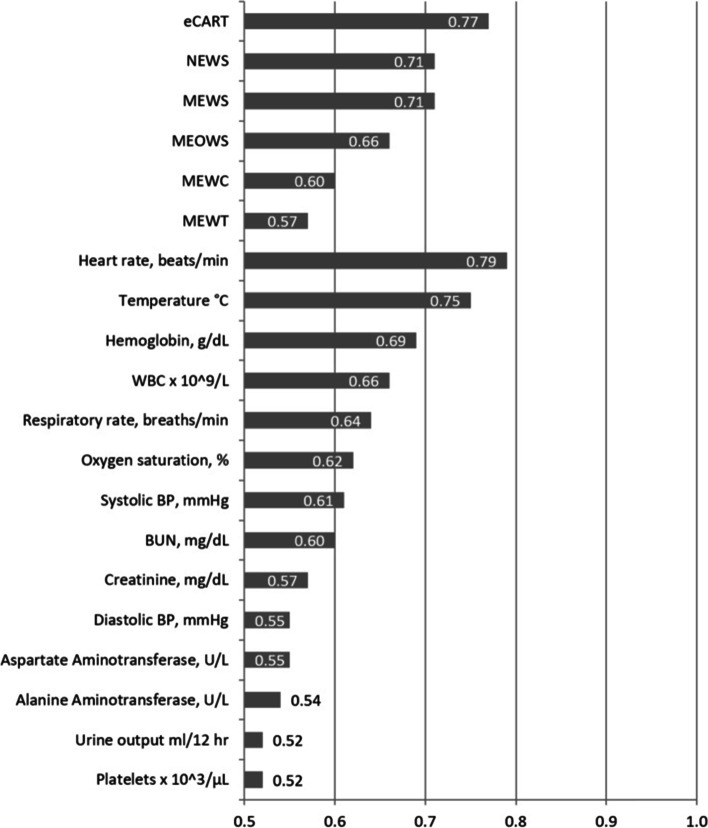
AUCs of early warning scores and individual variables for predicting infection

The performance of each scoring system, as well as the component vital signs and laboratory values for the secondary outcome of infection is shown in Fig. 3.

For predicting infection, eCART (AUC of 0.77; 95% CI: 0.75–0.78) had the highest discrimination, followed by MEWS (AUC of 0.71; 95% CI: 0.69–0.73) and NEWS (AUC of 0.71; 95% CI: 0.70–0.73). Heart rate (AUC 0.79; 95% CI: 0.77–0.80) in isolation performed better than any scoring system for this secondary outcome.

## Discussion

In this single center, retrospective study of 19,611 obstetric admission encounters, we compared the accuracy of general and obstetric scoring systems for identifying women on the ante- or postpartum floors who go on to be admitted to the ICU or die. Among the general risk scores, eCART had the highest discrimination, with improved accuracy over MEWS and NEWS across different risk thresholds. Although accuracy at specific thresholds was not always directly comparable, our results also suggest that the maternal early warning scores were less accurate than eCART but more accurate than MEWS and NEWS. Of the individual physiologic parameters, respiratory rate performed the best, followed by heart rate and systolic blood pressure, similar to results from general ward patients and post-operative patients [13, 19, 20]. For the secondary outcome of infection, eCART had the highest discrimination of the scoring systems analyzed despite not being developed for this purpose. However, heart rate alone was even more predictive than the scoring systems for this outcome. Overall these findings have important implications for the risk stratification of maternal hospitalized patients.

To our knowledge, this is the first study to investigate the accuracy of general early warning scores, maternal early warning scores, and a machine learning score (eCART) for predicting maternal outcomes. A major strength of our investigation is the large size of the population (> 19,000 admissions), which allowed us to study the performance of early warning algorithms for predicting ICU admission and maternal death, which are rare events. Some prior studies of maternal early warning systems used less severe definitions of morbidity or only investigated patients with specific conditions, limiting the strength of the conclusions that can be drawn regarding severe morbidity requiring ICU transfer and mortality [15, 16, 24]. Furthermore, some of the scoring systems studied have not been externally evaluated specifically in an unselected cohort of admitted ante- or postpartum patients [18]. Therefore, our findings provide important information regarding the expected performance of these scores when calculated over time in a general obstetric population.

Determining the accuracy of scoring systems for relevant outcomes is an important first step before performing interventional studies that use these scores. To date, few large studies have investigated the impact of maternal scoring systems on patient outcomes [18], although one notable study by Shields et al. found that implementing MEWT coupled with clinical treatment pathways decreased maternal morbidity [9]. Our findings suggest that if MEWT or MEWC are already implemented in a hospital system, then switching to a general early warning score, such as MEWS or NEWS, would likely result in decreased accuracy, while switching to eCART could improve accuracy. The choice between these systems should be based on the sensitivity–specificity trade-off, site-specific logistic considerations, and how many false alarms can be tolerated given resource constraints. Our results also suggest that MEWS and NEWS are suboptimal in patients on the antepartum or postpartum wards, and switching to one of the other systems may be warranted, with local analyses performed to confirm this if at all possible. Although eCART had the highest discrimination of all tools studied, its positive predictive values were still low due to the low rate of events. Future work to develop new machine learning models to predict deterioration and infection in obstetric populations could further improve accuracy, but large cohorts will be needed due to the low event rate in this population.

The primary limitations of our study are inherent to its retrospective, single-center design. Most importantly, improved score accuracy does not mean improved patient outcomes, and further study is needed to determine the impact of these scores on morbidity, mortality, and early provider recognition of women at risk for deterioration. In addition, we relied on a large electronic dataset to identify women on the antepartum and postpartum ward, and it is possible that this did not capture or accurately classify all admissions. Our study included patients only on the ward, so our results may not apply to patients in labor or the immediate postpartum period prior to transfer to the floor. Additionally, ante- or postpartum patients who are identified as being at risk for deterioration are often transferred to labor and delivery for more intensive monitoring, and our study only captures the subset of these patients who were transferred to the ICU or died within 24 h of transfer from the ward. Furthermore, our study is based on electronic health records and therefore may not generalize to settings where scores are calculated by hand at the bedside. We also were unable to capture subjective elements included in several of the obstetric scoring systems (e.g. nursing discomfort with patient status, patient with pre-eclampsia reporting a non-remitting headache) and thus our results may not be reflective of how these tools would perform with these elements included. The definition of infection has been validated but may not capture all clinically significant infections [21]. Finally, our study was performed retrospectively at a single center, and prospective validation in multiple centers would provide valuable information regarding the potential ability of eCART to detect clinically significant deterioration in the ante and postpartum population at a time when intervention has the potential to change outcomes.

## Conclusions

An early warning tool has the potential to identify patients who may be at risk for clinical deterioration at a time when early intervention has the potential to change outcomes [6, 9, 12]. While it was not possible to disentangle the relative impact of the detection tool in comparison to treatment pathways, we believe that optimizing early warning algorithms are an integral part of ongoing efforts to decrease maternal morbidity and mortality. We demonstrated that within the limitations of our retrospective study, eCART was the most accurate tool to predict deterioration and infection in our ante- and postpartum patient population, and that maternal early warning scores were more accurate than the MEWS and NEWS. As discussed in detail above, key limitations of our study include that we were unable to incorporate the subjective parameters included in some early warning systems, as well as the low overall event rate for ICU transfer or death in the ante and postpartum population. Institutional choice of an early warning system is complex and must be tailored to local needs and resources. Pairing accurate tools with evidence-based treatment pathways may help decrease the rising maternal mortality seen in the United States.

## Supplementary Information

**Additional file 1.** Component variables of the included early warning scores.

**Additional file 2.** Study Flow Diagram.

**Additional file 3.** Accuracy of early warning scores for predicting ICU transfer and/or death at different score thresholds.

## Data Availability

As the data are high granular in nature and contain potentially sensitive patient information, public sharing of the data would breach the University of Chicago’s IRB protocol requirements. Interested researchers may contact Mary Akel, University of Chicago, via email at makel@medicine.bsd.uchicago.edu for data access requests.

## Abbreviations

MEWS: Modified Early Warning Score
NEWS: National Early Warning Score
MEOWS: Modified Early Obstetric Warning System
MEWC: Maternal Early Warning Criteria
MEWT: Maternal Early Warning Trigger
eCART: Electronic Cardiac Arrest Triage
ICU: Intensive Care Unit
IV: Intravenous
AUC: Area Under the receiver operating characteristic Curve
