# Supplementary material for: Comparison of early warning scores for predicting clinical deterioration and infection in obstetric patients
Source: BMC Pregnancy Childbirth. 2022 Apr 6;22:295. doi: 10.1186/s12884-022-04631-0 (PMC8988389; doi:10.1186/s12884-022-04631-0)
Supplement: Supplementary file 1 — Additional file 1. Component variables of the included early warning scores. [file 12884_2022_4631_MOESM1_ESM.docx]

**Additional File 1:** Component variables of the included early warning scores.

| Early warning score | Component variables |
| --- | --- |
| MEWS^a^ | Heart rate, respiratory rate, blood pressure, temperature, mental status |
| NEWS^b^ | Heart rate, respiratory rate, blood pressure, temperature, mental status, oxygen saturation, supplemental oxygen |
| eCART^c^ | Heart rate, respiratory rate, blood pressure, temperature, mental status, oxygen saturation, supplemental oxygen, albumin, alkaline phosphatase, bilirubin, BUN,^d^ calcium, chloride, CO2, creatinine, serum glucose, hemoglobin, platelet count, potassium, AST,^e^ sodium, total protein, WBC,^f^ age, ICU^g^ stays, ward hours |
| MEOWS^h^ | Heart rate, respiratory rate, blood pressure, temperature, mental status, oxygen saturation, pain scale |
| MEWC^i^ | Heart rate, respiratory rate, blood pressure, temperature, oxygen saturation mental status, patient with preeclampsia reporting a non-remitting headache or shortness of breath |
| MEWT^j^ | Heart rate, respiratory rate, blood pressure, temperature, mental status, oxygen saturation, supplemental oxygen, nursing clinically uncomfortable with patient status |

a: Modified Early Warning Score

b: National Early Warning Score

c: electronic Cardiac Arrest Triage

d: Blood urea nitrogen

e: Aspartate aminotransferase

f: White blood cell count

g: Intensive Care Unit

h: Modified Early Obstetric Warning System

i: Maternal Early Warning Criteria

j: Maternal Early Warning Trigger
