# Supplementary figures and images for: Comparison of early warning scores for predicting clinical deterioration and infection in obstetric patients
Source: BMC Pregnancy Childbirth. 2022 Apr 6;22:295. doi: 10.1186/s12884-022-04631-0 (PMC8988389; doi:10.1186/s12884-022-04631-0)

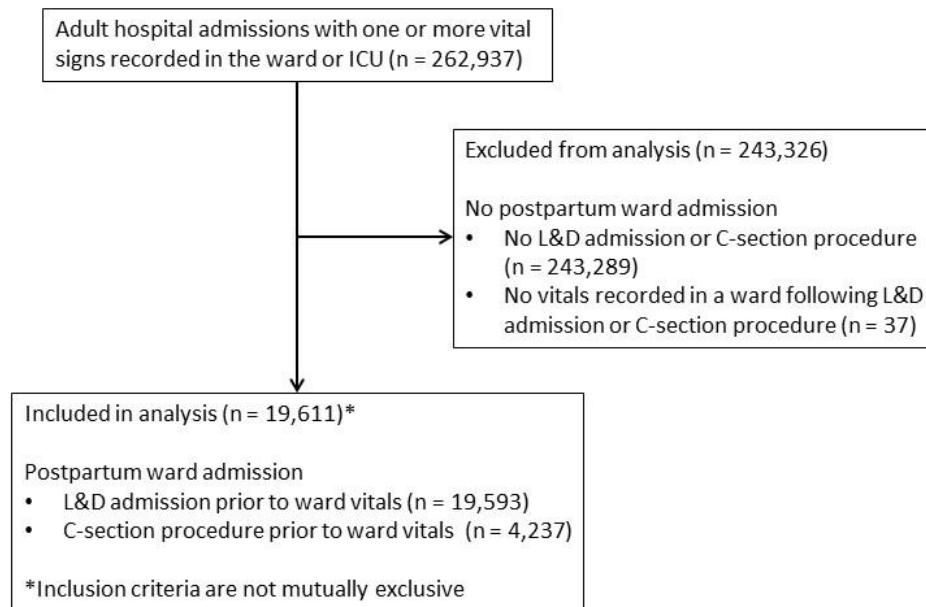

#### **Additional File 2: Study Flow Diagram**

Flow diagram describing patients included in the study.

Supplement: Supplementary file 2 — Additional file 2. Study Flow Diagram. [file 12884_2022_4631_MOESM2_ESM.pdf]
