# Supplementary material for: Comparison of early warning scores for predicting clinical deterioration and infection in obstetric patients
Source: BMC Pregnancy Childbirth. 2022 Apr 6;22:295. doi: 10.1186/s12884-022-04631-0 (PMC8988389; doi:10.1186/s12884-022-04631-0)
Supplement: Supplementary file 3 — Additional file 3. Accuracy of early warning scores for predicting ICU transfer and/or death at different score thresholds. [file 12884_2022_4631_MOESM3_ESM.docx]

**Additional File 3:** Accuracy of early warning scores for predicting ICU transfer and/or death at different score thresholds.

| Early warning score | Threshold | Sensitivity | Specificity | PPV^a^ | NPV^b^ |
| --- | --- | --- | --- | --- | --- |
| MEWS^c^ | ≥2 | 59.5% | 69.2% | 0.3% | 99.9% |
|  | ≥3 | 44.1% | 93.2% | 1.0% | 99.9% |
|  | ≥4 | 27.5% | 98.6% | 3.0% | 99.9% |
|  | ≥5 | 14.8% | 99.7% | 6.7% | 99.9% |
| NEWS^d^ | ≥3 | 55.9% | 83.0% | 0.5% | 99.9% |
|  | ≥4 | 43.4% | 92.3% | 0.9% | 99.9% |
|  | ≥5 | 33.7% | 97.0% | 1.7% | 99.9% |
|  | ≥6 | 25.9% | 98.9% | 3.4% | 99.9% |
|  | ≥7 | 18.9% | 99.6% | 7.2% | 99.9% |
| MEOWS^e^ | Two yellow triggers | 20.2% | 91.2% | 0.4% | 99.9% |
|  | One red trigger | 51.5% | 94.9% | 1.5% | 99.9% |
|  | Two yellow triggers and/or one red trigger | 61.4% | 86.9% | 0.7% | 99.9% |
| MEWC^f^ | One abnormal parameter | 53.3% | 88.9% | 0.7% | 99.9% |
| MEWT^g^ | Two non-severe triggers | 4.9% | 99.3% | 1.0% | 99.9% |
|  | One severe trigger | 27.5% | 98.8% | 3.5% | 99.9% |
|  | Two non-severe triggers and/or one severe trigger | 31.4% | 98.1% | 2.5% | 99.9% |
| eCART^h^ | ≥0.0035 | 67.3% | 87.4% | 0.8% | 99.9% |
|  | ≥0.0038 | 64.0% | 89.4% | 0.9% | 99.9% |
|  | ≥0.0040 | 61.9% | 90.6% | 1.0% | 99.9% |
|  | ≥0.0042 | 58.6% | 91.6% | 1.1% | 99.9% |
|  | ≥0.0045 | 55.5% | 93.0% | 1.2% | 99.9% |
|  | ≥0.0046 | 52.9% | 93.4% | 1.2% | 99.9% |
|  | ≥0.0047 | 52.0% | 93.8% | 1.3% | 99.9% |
|  | ≥0.0051 | 49.0% | 95.0% | 1.5% | 99.9% |
|  | ≥0.0053 | 45.6% | 95.6% | 1.6% | 99.9% |
|  | ≥0.0060 | 40.9% | 97.0% | 2.1% | 99.9% |
|  | ≥0.0069 | 35.5% | 98.1% | 2.9% | 99.9% |
|  | ≥0.0074 | 33.7% | 98.5% | 3.4% | 99.9% |
|  | ≥0.0075 | 32.9% | 98.6% | 3.5% | 99.9% |
|  | ≥0.0079 | 31.9% | 98.8% | 4.1% | 99.9% |
|  | ≥0.0090 | 27.5% | 99.3% | 5.6% | 99.9% |
|  | ≥0.0108 | 23.6% | 99.6% | 9.0% | 99.9% |

a: Positive Predictive Value

b: Negative Predictive Value

c: Modified Early Warning Score

d: National Early Warning Score

e: Modified Early Obstetric Warning Score

f: Maternal Early Warning Criteria

g: MEWT: Maternal Early Warning Tool

h: electronic Cardiac Arrest Triage
